# Complications of Peripherally Inserted Central Venous Catheters: A Retrospective Cohort Study

**DOI:** 10.1371/journal.pone.0162479

**Published:** 2016-09-02

**Authors:** Paula Parás-Bravo, María Paz-Zulueta, Raquel Sarabia-Lavin, Francisco Jose Amo-Setién, Manuel Herrero-Montes, Encarnación Olavarría-Beivíde, Mercedes Rodríguez-Rodríguez, Blanca Torres-Manrique, Carlos Rodríguez-de la Vega, Vanesa Caso-Álvarez, Laura González-Parralo, Francisco Manuel Antolín-Juárez

**Affiliations:** 1 Departamento de Enfermería, Universidad de Cantabria, Santander, Cantabria, España; 2 Servicio Cántabro de Salud, Santander, Cantabria, España; Universite de Bretagne Occidentale, FRANCE

## Abstract

**Background and Aim:**

The use of venous catheters is a widespread practice, especially in oncological and oncohematological units. The objective of this study was to evaluate the complications associated with peripherally inserted central catheters (PICCs) in a cohort of patients.

**Materials and Methods:**

In this retrospective cohort study, we included all patient carrying PICCs (n = 603) inserted at our institute between October 2010 and December 2013. The main variables collected were medical diagnosis, catheter care, location, duration of catheterization, reasons for catheter removal, complications, and nursing care. Complications were classified as infection, thrombosis, phlebitis, migration, edema, and/or ecchymosis.

**Results:**

All patients were treated according to the same “nursing care” protocol. The incidence rate of complications was two cases per 1000 days of catheter duration. The most relevant complications were infection and thrombosis, both with an incidence of 0.17 cases per 1000 days of the total catheterization period. The total average duration of catheterization was 170 days [SD 6.06]. Additionally to “end of treatment” (48.42%) and “exitus”, (22.53%) the most frequent cause of removal was migration (displacement towards the exterior) of the catheter (5.80%).

**Conclusions:**

PICCs are safe devices that allow the administration of long-term treatment and preserve the integrity of the venous system of the patient. Proper care of the catheter is very important to improve the quality life of patients with oncologic and hematologic conditions. Therefore, correct training of professionals and patients as well as following the latest scientific recommendations are particularly relevant.

## Introduction

The use of central venous lines is justified by the need to instill certain treatments in a continuous manner and in large doses. These catheters are essential to modern medical practice, and they are especially necessary when the treatments are vesicants or irritating, such as chemotherapy and some antibiotics. Central venous catheters are even indicated in the absence of peripheral venous access or when this is very damaged [1].

At present, central venous catheter use has become widespread in medical specialties including critical care, dialysis, nutritional support, and oncology. The most frequent indications for their use are the administration of chemotherapy, parenteral nutrition, and treatments considered vesicants or irritating [2, 3]. Typically, these devices are used for patients with complicated venous access, impairment of the lymphatic system, pain during infusion, and fear of venipuncture. Additionally, the placement of a central venous catheter for outpatient administration of drugs is necessary in patients with continuous perfusion of chemotherapy. Otherwise, the patient would require hospital admission for administration through the peripheral venous line. Thanks to the placement of a central venous catheters in such cases, the latter circumstance has been practically eliminated at our hospital.

**Fig 1:**
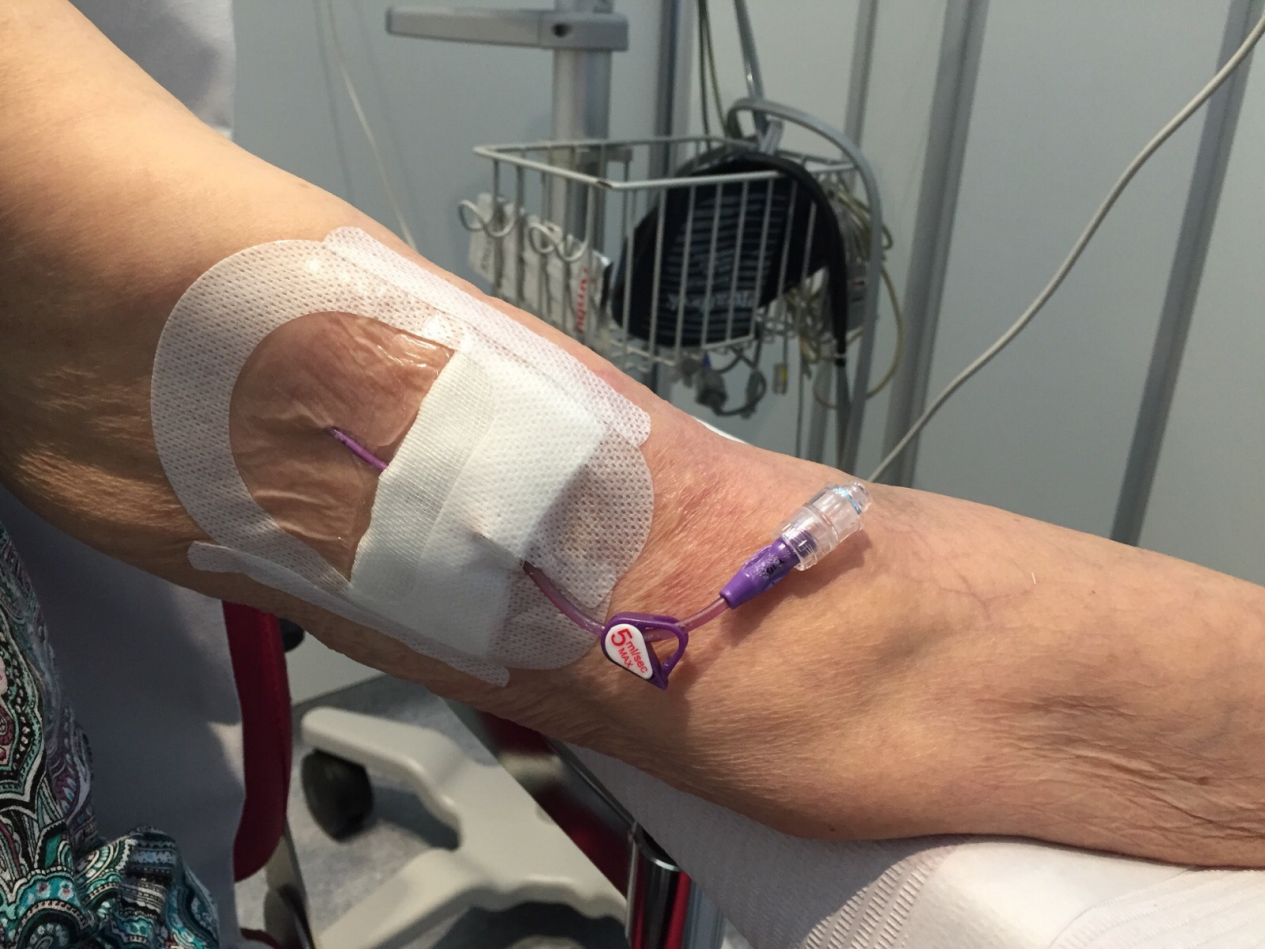
Long-term peripherally inserted central catheters (PICCs).

For the administration of large volumes of chemotherapy drugs, oncohematological patients need catheters that flow into a vein with sufficient flow and caliber. Long-term peripherally inserted central catheters (PICCs) (Fig 1) that flow into the lower third of the vena cava meet these criteria and reduce damage of the peripheral vascular system, which favors the maintenance of their integrity [4].

Using a PICC, the risk of extravasation is markedly reduced, which is particularly relevant for the administration of drugs of a vesicant or irritating nature such as some cytostatics. Although this type of venous port represents a safe method of permanent access with a low rate of complications [5], some complications may still occur. Of all complications, infection and thrombosis are the most important owing to their relevance and clinical consequences for oncohematological patients. Such complications may be associated with increased morbidity and depending on the baseline situation of the patient, may even increase mortality. Deep vein thrombosis is the most common complication in patients receiving chemotherapy, and the risk is increased in patients with diabetes, chronic obstructive pulmonary disease, or presence of metastases [6]. The risk of infection is greater for oncohematological patients because the treatment and the pathology itself are associated with periods of immunosuppression [7].

The general objective of this study was to evaluate the incidence rate of complications associated with PICCs in a hospital in northern Spain with a cohort of mostly oncohematological patients.

## Materials and Methods

### Study design

This was a retrospective longitudinal cohort study.

### Setting

This study was conducted in the Day Hospital, Marqués de Valdecilla University Hospital, in Cantabria, a region in northern Spain, between October 2010 and December 2013.

### Participants

The inclusion criteria were as follows: male or female patients, ≥18 years of age and carriers of PICCs during the study period. Data collection was retrospective.

The study population included all the patients’ carriers of PICCs during the study period, who were treated at our institution. The final study population was 603 patients.

### Variables and Measures

The following baseline data were obtained for each patient: date of birth, sex, medical diagnosis, nursing care of the catheter, the location of the catheter, duration of catheterization (days), reasons for catheter removal, and complications.

Catheters were inserted by trained nursing staff, by sterile and ultrasound-guided technique (two-dimensional ultrasound imaging). The PICCs were placed in the middle third of the upper arm, above the antecubital fossa, in either the cephalic or basilic vein. We chose a vein of a caliber proportional to the caliber of the catheter. The distal tip of the catheter was placed in the lower third of the superior vena cava, which was later confirmed by chest radiograph [8]. We used PowerPicc®, a polyurethane, 5-Fr diameter, single-lumen catheter. Fixation and stabilization was achieved with a sterile latex-free device designed for this purpose (StatLock PICC Plus Stabilization Device®).

The “Nursing protocol” was applied to all patient carriers of PICCs and consisted of weekly sterile dressing with transparent bandages and cleaning with chlorhexidine solution according to the recommendations of the United States Centers for Disease Control and Prevention (CDC) [9]. When the fixing device was deteriorated, it was replaced with a new one. The catheters were sealed with heparin after use [10, 11].

The duration of catheterization was calculated as the difference between the date of insertion and the date of removal. This date was handled in the analyses like a continuous quantitative variable.

Complications were categorized as infection, thrombosis, phlebitis, migration, edema, and/or ecchymosis. The criteria for defining the occurrence of “infection” were the Central Line Associated Blood Stream Infection (CLABSI) [9].

Symptoms of thrombosis were pain, swelling, erythema and vein blockage by a blood clot (thrombus) with subsequent lack of permeability [12]. The criteria for the diagnosis of “thrombosis” were the presence of symptoms and final confirmation by ultrasound.

Phlebitis was defined as irritation of the venous endothelium by the catheter. Its symptoms were similar to those of thrombosis, but the catheter remained permeable because there was no venous blockage: pain, tenderness, swelling, erythema, palpable venous cord, purulent discharge, and warmth of the area [13]. The criteria for a diagnosis of “phlebitis” were the presence of symptoms and final confirmation by ultrasound.

The criterion for catheter migration was outward displacement of the catheter by more than 2 cm (displacement towards the exterior). The diagnostic criterion for edema was swelling caused by fluid accumulation in the arm near the catheter insertion point. The criterion for the diagnosis of ecchymosis was the presence of a bruise near the catheter insertion point. The catheter was considered to have a “lumen occlusion” if there was total occlusion, occlusion to flush only, occlusion for aspiration only, or subjective difficulty with flushing or aspiration only, if the vein was not damaged, inflamed or obstructed and fibrinolytic treatment was required [8, 10].

### Study limitations

In retrospective studies based on secondary information (medical records), one of the main limitations is that the data obtained from computer records may be subject to bias by the researcher who collected the information. In this study, the personnel of the unit responsible for the care of the PICCs was trained for data collection by standardized training sessions with the objective of reducing the interobserver differences as much as possible.

Additionally, another study limitation is that the nursing protocol has been currently updated. At the present time, it is recommended to clean the catheter with saline [8]. However, this study was conducted between October 2010 and December 2013, and our protocol at that time consisted of the prophylactic use of heparin [11, 14].

### Statistical analyses

For the descriptions of the variables, frequency distributions, averages with 95% confidence intervals (95% CI), standard deviations, and ranges were calculated. The normal distribution of data was assessed using the Kolmogorov-Smirnov test. The cumulative incidence and the incidence density rates per 1000 days of catheter use of the various complications were calculated. For comparison of proportions, a chi-square test was performed, using Fisher’s exact test when necessary. Nonparametric tests were used, such as the Mann-Whitney U-test, for the comparison of averages, and the Kendall test was used in the correlation studies when the variables did not have a normal distribution. For the study of associated independent factors, an automated stepwise forward multiple logistic regression model was used. The alpha error was set at 0.05, and all p-values were bilateral. All statistical analyses were conducted using IBM SPSS Statistics version 22.0 (IBM Corp., Armonk, NY).

### Ethics Statement

Approval of the research protocol was obtained on February 25, 2014 from the Clinical Research Ethics Committee of Cantabria before the acquisition of data. Patient records/date were anonymized and de-identified prior to analysis. Because the data were analyzed anonymously, patient consent was not necessary. We obtained the consent of the Ethics Committee and authorization from the hospital to perform this study.

## Results

A total of 603 patient carriers of PICCs aged between 18 and 95 years (Table 1) were analyzed. Regarding sex, 54.73% were female, and 45.27% were male.

**Table 1:** Age distribution of patients with peripherally inserted central catheters. Cantabria (Spain): patient carriers of peripherally inserted central catheters, 2010–2013.

AGE RANGE (years)	n = 603	(%)
**10–19**	2	0.33
**20–29**	13	2.16
**30–39**	43	7.13
**40–49**	92	15.26
**50–59**	161	26.70
**60–69**	186	30.85
**70–79**	76	12.60
**80–89**	23	3.81
**90–99**	7	1.16

With regard to diagnosis, 93.86% (n = 566) of the patients had been diagnosed with an oncologic or oncohematologic disease, while the remaining patients presented with diseases of other etiologies (Table 2). The proportion of oncological diseases was higher than that of oncohematological diseases; the former constituted nearly 74% of the patients (n = 466).

**Table 2:** Distribution diagnosis of patients with peripherally inserted central catheters. Cantabria (Spain): patient carriers of peripherally inserted central catheters, 2010–2013.

MEDICAL DIAGNOSIS	n = 603	(%)
**Colorectal cancer**	149	24.70
**Breast cancer**	106	17.58
**Non Hodgkin lymphoma**	49	8.13
**Pancreas cancer**	44	7.30
**Sarcoma**	33	5.47
**Leukemia**	31	5.14
**Hodgkin lymphoma**	25	4.15
**Ovarian cancer**	23	3.81
**Lung cancer**	22	3.65
**Bladder cancer**	15	2.49
**Gastric cancer**	15	2.49
**Infection**	13	2.16
**Ischemia chronicle**	12	2.00
**Head neck cancer**	9	1.49
**Not collected**	8	1.33
**Malabsorption syndrome**	7	1.16
**Anal cancer**	5	0.83
**Multiple myeloma**	5	0.83
**Testicles cancer**	4	0.66
**Esophagus cancer**	4	0.66
**Anemia**	4	0.66
**Cervix cancer**	4	0.66
**Prostate cancer**	2	0.33
**Melanoma**	2	0.33
**Prostate cancer**	2	0.33
**Kidney cancer**	2	0.33
**Myelodysplastic syndrome**	2	0.33
**Biliary cancer**	1	0.16
**Brain cancer**	1	0.16
**Myelofibrosis**	1	0.16
**Merkeloma**	1	0.16
**Thymoma**	1	0.16

The average duration of catheterization was 171.20 days [SD 6.06] with a minimum of 2 days and a maximum of 882 days. All the catheters were located in the middle third of the upper arm, above the antecubital fossa: 34.99% (n = 211) were placed in the right basilic vein, 56.05% (n = 338) were placed in the left basilic vein, 2.98% (n = 18) were placed in the right cephalic vein, and 5.97% (n = 36) were placed in the left cephalic vein.

At least one complication occurred in 38.14% of the patients (n = 230), whereby the incidence of complications was two complications per 1000 days of the total duration of catheterization. The individual analysis of complications is presented in Table 3.

**Table 3:** Analysis of complications of peripherally inserted central catheters. Cantabria (Spain): patient carriers of peripherally inserted central catheters, 2010–2013.

COMPLICATION	n	(%)	95%IC	Incidence rate^a^	Day of onset^b^
**Infection**	19	3.15	1.67–4.63	0.17	114.26 [22.21]
**Thrombosis**	20	3.32	1.81–4.83	0.17	28.90 [9.12]
**Phlebitis**	43	7.13	4.99–9.27	0.38	2.23 [0.21]
**Migration**	79	13.10	10.32–15.88	0.69	163.75 [14.15]
**Edema**	5	9.12	10.32–15.88	0.50	28.16 [9.36]
**Ecchymosis**	111	18.40	15.23–21.58	1.93	3.96 [0.42]
**Lumen occlusion**	267	44.27	40.23–48.32	2.32	76.48 [73.66]

^a^ Per 1.000 days of use.

^b^ Mean [SD]. Abbreviations: IC, confidence interval; SD, standar deviation.

Additionally, 3.15% (95% CI 1.67–4.63%) (n = 19) of patients presented an infection profile. Complication onset after catheterization occurred at an average of 114.26 [SD 22.21] days. The incidence rate of complications was 0.17 cases per 1000 days for the total duration of catheterization.

Thrombosis occurred in 3.32% (95% CI 1.81–4.83%) (n = 20) of the patients, at an average onset of 28.90 [SD 9.12] days of the total duration of catheterization. The incidence rate of thrombosis was 0.17 cases per 1000 days of the total duration of catheterization. In 13 of the patients, a fibrinolytic agent was used at least once. In all cases, the thrombosis event was local.

In 7.13% (95% CI 4.99–9.27%) (n = 43) of patients, phlebitis was registered at an average onset of 2.23 [SD 0.21] days after catheter insertion. The incidence rate was 0.38 cases per 1000 days of the total catheter days.

In 13.10% (95% CI 10.32–15.88%) (n = 79) of the catheters, at least one episode of migration was registered at an average onset of 163.75 [SD 14.15] days after insertion. The latest case occurred on day 605. The incidence rate was 0.69 cases per 1000 days of the total duration of catheterization.

In 9.12% (n = 55) (95% CI 10.32–15.88%) of patients, edema onset occurred at an average onset of 28.16 [SD 9.36] days of the total duration of catheterization. In 20 patients, this complication occurred on the day following catheter insertion. In contrast, four cases were registered between days 237 and 282 of catheterization. The incidence rate was 0.50 cases per 1000 days of the total duration of catheterization.

In 18.40% (n = 111) (95% CI 15.23–21.58%) of patients, an ecchymosis was produced at an average onset of 3.96 [SD 0.42] days of the total duration of catheterization, although a high frequency of cases (n = 47) was noted on the day following catheter insertion. This complication showed the highest incidence rate, with 1.93 cases per 1000 days of the total duration of catheterization.

In 44.27% (95% CI 40.23–48.32) (n = 267) of the patients, a fibrinolytic therapy was used at least once because of catheter lumen occlusion. The incidence rate was 2.32 cases per 1000 days of the total duration of catheterization. The average onset was at 76.48 [SD 73.66] days after insertion.

No significant differences were identified regarding sex, age, medical diagnosis, and location of the catheter with respect to infection, thrombosis, phlebitis, migration, edema, ecchymosis, and/or lumen occlusion. No variable remained significant in the multivariable model (Table 4).

**Table 4:** Presence of complications regarding sex, age, diagnosis, and localization of the peripherally inserted central catheters. Cantabria (Spain): patient carriers of peripherally inserted central catheters, 2010–2013.

VARIABLES		TOTAL	COMPLICATIONS^a^	
			N	(%)
**SEX**				
	**Men**	273	99	16.41
	**Women**	330	131	21.72
***p 0*.*0388***^***b***^				
**AGE**				
	**>65 years**	180	65	10.78
	**<65 years**	423	165	27.36
***p 0*.*503***^***b***^				
**MEDICAL DIAGNOSIS**				
	**Oncohematologic**	114	36	5.97
	**Oncologic**	445	179	29.68
	**Other / not collected**	44	15	2.49
***p 0*.*238***^***b***^				
**LOCATION**				
	**Basilic right**	211	72	11.94
	**Basilic left**	338	134	22.22
	**Cephalic right**	18	6	0.99
	**Cephalic left**	36	18	2.98
***p* 0.252**^***b***^				
**DURATION**				
	**>150 days**	298	130	21.56
	**<150 days**	305	100	16.58
***p 0*.*006***^***b***^				

^a^Presence of at least one of the following complications: Infection, thrombosis, phlebitis, migration, edema, ecchymosis and/or lumen occlusion.

^b^Pearson's chi-squared test.

No correlation was found (correlation coefficient 0.0023; p = 0.379) between age and duration, location, and migration (displacement towards the exterior) of the catheter. However, the complication rate was significantly higher when the total duration of catheterization was or exceeded 150 days (p = 0.006), appearing in 43.62% (n = 130) of cases compared with 32.78% (n = 100).

Regarding the reason for removal of the catheter, in 48.42% (n = 292) of patients, the reason was “end of treatment”; other reasons included “exitus” in 22.55% (n = 136), “migration” in 5.80% (n = 35), “infection” in 4.14% (n = 25), “lumen occlusion” in 4.14% (n = 25), “replacement” in 1.99% (n = 12), and “thrombosis/thrombophlebitis/phlebitis” in 1.82% (n = 11) of patients. In 11.11% (n = 67) of the patients, the reason was not recorded or the cause was unknown (Table 5).

**Table 5:** Reasons for catheter removal of peripherally inserted central catheters. Cantabria (Spain): patient carriers of peripherally inserted central catheters, 2010–2013. Abbreviations: IC, confidence interval.

CAUSE OF WITHDRAWAL	n = 603	(%)	95%IC
**End of treatment**	292	48.42	44.35–52.50
**Exitus**	136	22.53	19.13–25.97
**Migration**	35	5.80	3.85–7.75
**Infection**	25	4.14	2.47–5.82
**Lumen Occlusion**	25	4.14	2.47–5.82
**Replacement**	12	1.99	0.79–3.18
**Phlebitis or thrombosis**	11	1.82	0.67–2.97
**Not collected**	67	11.11	8.52–13.70

## Discussion

The care of PICCs is a common practice for nursing professionals [15]; however, on some occasions, our actions are not supported by evidence-based practice [16–19]. Some studies agree that the difference in the rate of complications is directly related to the knowledge of nursing personnel regarding catheter placement and care. The rate of complications is significantly lower when training is adequate [4, 20, 21]. In fact, the CDC states in its guide for the care of central venous catheters that training of personnel is a fundamental aspect of preventing infections with a level of evidence of 1A [9]. Therefore, insertion and care of these catheters constitutes a priority area for research in nursing, not only to reduce the adverse effects but also to improve the patient experience [17].

Compared with other published studies, our rates of infection and thrombosis were lower (0.17 cases per 1000 days of the total catheter days) and showed cumulative incidences of 3.15% and 3.32%, respectively.

With regard to infection, in 2013, the National Healthcare Safety Network reported the incidence rate of infection of laboratory-confirmed temporary catheter use in oncohematological patients, which increased to 0.25 per 1000 days of total PICC duration [22]. Chopra et al. [23] examined this matter in a systematic review with meta-analysis. After reviewing 57,250 patients from 23 studies, they concluded that the incidence rate of bacteremia (collected in 13 of the studies) in PICC patients was 0.91% (95% CI 0.46–1.79) cases per 1000 days of total PICC duration. Another study conducted in a French university hospital with 222 patients reported an overall infection rate of 2.35 per 1000 days of catheter use with an incidence of 0.86 cases per 1000 days of total PICC duration [20]. Baxi et al. [24] found 57 cases of bacteremia when studying 609 patients with an incidence rate of 2.69/1000 PICCs/day. However, none of these studies exclusively included oncohematological patients. The study by Mollee et al. [25] was the most similar to our own. In their study, they retrospectively evaluated 727 oncohematological patients and found an incidence rate of infection of 2.5 per 1000 days of catheter use. Additionally, Aw et al. [6] studied cancer patients with a cumulative infection incidence rate of 5.6% compared with our rate of 3.15%.

With respect to thrombosis, our results yielded an incidence rate lower than expected with 0.17 cases per every 1000 days of PICC use and a cumulative incidence of 3.32%. Baxi et al. [24] also assessed this variable and obtained an incidence rate of 1.23/1000 PICC days. The cumulative incidence was approximately 8.4% in a study conducted in neurological patients in intensive care, in which 431 patients with PICCs [26] were studied. However, Aw et al. [6] studied a population of only 340 cancer patients of which 19 presented infection, comprising 5.6% (95% CI (3.06–8.06) of the patient total. Their patients resembled our patient sample in terms of characteristics; nonetheless, the complication rate in our study was lower.

Malpositioning of the catheter tip can cause difficulties with blood withdrawal and contribute to catheter occlusion. Therefore, it is very important to check its position radiologically to reduce this complication [8]. It is necessary to avoid occlusions because these are also associated with infection and thrombosis [10]. Further, placement of catheters above the antecubital fossa diminishes the likelihood of thrombophlebitis [8].

It is worth mentioning that the duration of catheterization in our study was much longer than those in the previously mentioned studies [20, 22, 23]. This was even true in some cases of catheterization longer than 2 years, with an average of 6 months. This may be attributable to the low rate of complications, which thereby contributed to maintaining the catheter for a longer period of time. This is especially relevant for oncohematological patients because they can conclude their treatment without changing devices. However, it must also be noted that the rate of complications in our study began to increase after 150 days of catheterization, increasing to a rate of 43.62% (n = 13). We could not compare these data because we were unable to find a similar study that achieved a duration of catheterization similar to ours.

The heterogeneity between the study results is justified by the differences between patients and the care applied to the catheters. There is significant variability in the recommendations for their care [27, 28]. In our case, following the latest recommendations at the time of the study [9], the insertion was performed in an echo-guided manner, which reduces complications [29]. Additionally, patient handling was performed in a sterile and systematic manner almost exclusively by the same functional unit with personnel trained for this purpose.

With respect to the “nursing protocol” that was applied to all patients by professionals trained in PICC care in a systematic manner, we consider that this aspect is partly responsible for the low rate of complications. However, currently, washing the catheter with saline is recommended, and therefore, our “nursing protocol” needs to be updated [8]. Perhaps with these new recommendations, we can decrease the incidence rate of lumen occlusion.

Finally, we consider that the catheter material also influenced our incidence of complications. In our case, the material used was polyurethane, and this is relevant because polyurethane PICC lines have been associated with lower rates of infection, dislodgment, thrombus, and rupture complications [30].

One of the main limitations of retrospective studies based on secondary information (medical records), could be the low quality of the information, owing either to incomplete records or a lack of agreement among the different records. In our study, information about the main variables was collected in more than 90% of patients. We were only unable to find the catheter removal cause in 67 cases. Another aspect to consider is the lack of availability of other potential confounders (e.g., presence of metastases), which are unavailable from the secondary registers used in our study.

## Conclusions

In conclusion, the complication incidence rate was very low, considering that complications included ecchymosis, edema, and lumen occlusion. The latter were transient and not serious complications that did not require removal of the catheter. We will continue our line of research and study of the relationship between our nursing care and the appearance of complications.

## Supporting Information

S1 Data Study database. (XLS)

S1 Checklist Strobe Checklist. (PDF)
