# Supplementary material for: Complications of Peripherally Inserted Central Venous Catheters: A Retrospective Cohort Study
Source: PLoS One. 2016 Sep 2;11(9):e0162479. doi: 10.1371/journal.pone.0162479 (PMC5010186; doi:10.1371/journal.pone.0162479)
Supplement: S1 Checklist — (PDF) [file pone.0162479.s002.pdf]

Item 1

a) Yes, “Complications of peripherally inserted central venous catheters: A retrospective cohort study”.
b) Yes, the objective of study was determine the complications associated with peripherally inserted central catheters (PICCs) in a cohort of patients through a retrospcetive study. The abstract is estructured with background and aim, methods, results and conclusions.

Item 2

Yes

Item 3

Yes, but we don’t include hypotheses.

Item 4

Yes

Item 5

Yes, In the Day Hospital, Marqués de Valdecilla University Hospital, in Cantabria, a region in northern Spain, between October 2010 and December 2013. Data collection was retrospective.

Item 6

a) Yes, The inclusion criteria were: male or female, ≥18 years old and carriers of PICCs during the study period.

The study population was all the patients carriers of PICCs during the study period, who were attended in In the Day Hospital in Cantabria, a region in northern Spain. The final study population were 603 patients.

b) Not applicable in our study

Item 7

Yes, The main study variables were: Date of birth, sex, medical diagnosis, nursing care of the catheter, location of the catheter, the total catheter days, reasons for withdrawal, and complications.

Item 8

Yes, The total catheter days were estimated like difference between the date of insertion and the date of withdrawal. This date was handled in the analyses like continuous quantitative variable.

The complications were categorized as infection, thrombosis, phlebitis, migration, edema, and/or ecchymosis.

The criteria for defining the occurrence of “infection” were the Central Line Associated Blood Stream Infection (CLABSI) (O’Grady, 2011).

The criteria for the diagnosis of “thrombosis” were initially the symptomatology and, finally, was confirmed by ultrasound.

The criteria for diagnosis of "phlebitis” (irritation of the venous endothelium by the catheter) were initially the symptomatology and, finally, was confirmed by ultrasound.

The criteria for determining the "migration" was when the catheter had moved outward more of two centimetres (displacement towards the exterior).

The criteria for the diagnosis of “edema” were swelling caused by fluid accumulation in the arm near the catheter insertion point.

The criteria for the diagnosis of " ecchymosis " were the presence of bruise near the catheter insertion point.

The catheter was considered with "lumen occlusion" when the catheter was not suitably permeable and it did not respond to fibrinolytic therapy or if fibrinolytic or anticoagulation therapy was contraindicated (Schiffer, 2013).

Item 9

Yes

Item 10

Yes, The study population was all the patients carriers of PICCs during the study period, who were attended in In the Day Hospital in Cantabria, a region in northern Spain. The final study population were 603 patients.

Item 11

Yes, see Statistical Analyses

Item 12

Yes, see "Statistical Analyses"

Item 13

a) Yes.

b) Not applicable in our study

c) Not applicable in our study

Item 14

a) Yes, see Table 4.

b) Yes, see Table 4.

c) Yes, see Table 4.

Item 15

Yes, see Table 3, 4 and 5.

Item 16

Yes, see Table 1-5.

Item 17

Yes.

Item 18

Yes.

Item 19

Yes. In retrospective studies based on secondary information (records), one of the main limitations could be the low quality of the information, owing either to incomplete records or a lack of agreement among the different

records. In our study, information about the main variables was collected in more than 90% of patients. Only, we have 67 missing in the variable “cause of withdrawal”.Another aspect to consider is the lack of availability of other potential confounders (presence of metastases), which are unavailable from the secondary registers used in our study.

Item 20

Yes.

Item 21

Yes.

Item 22

Not applicable in our study
